# Complex roles for proliferating cell nuclear antigen in restricting human cytomegalovirus replication

**DOI:** 10.1128/mbio.00450-25

**Published:** 2025-03-25

**Authors:** Pierce Longmire, Olivia Daigle, Sebastian Zeltzer, Matias Lee, Marek Svoboda, Marco Padilla-Rodriguez, Carly Bobak, Giovanni Bosco, Felicia Goodrum

**Affiliations:** 1 Graduate Program in Molecular Medicine, University of Arizona 8041 , Tucson, Arizona, USA; 2 Department of Immunobiology, University of Arizona 242724 , Tucson, Arizona, USA; 3 BIO5 Institute, University of Arizona BIO5 Institute 124486 , Tucson, Arizona, USA; 4 Department of Molecular and Systems Biology, Dartmouth Geisel College of Medicine 198508 , Hanover, New Hampshire, USA; 5 Research Computing and Data Services, Information, Technology, and Consulting, Dartmouth College 3728 , Hanover, New Hampshire, USA; 6 Microscopy Shared Resource, The University of Arizona Cancer Center 613590 , Tucson, Arizona, USA; Princeton University, Princeton, New Jersey, USA

**Keywords:** herpesvirus, cytomegalovirus, DNA Damage, DNA repair, PCNA, genome analysis

## Abstract

DNA viruses at once elicit and commandeer host pathways, including DNA repair pathways, for virus replication. Despite encoding its own DNA polymerase and processivity factor, human cytomegalovirus (HCMV) recruits the cellular processivity factor, proliferating cell nuclear antigen (PCNA) and specialized host DNA polymerases involved in translesion synthesis (TLS) to replication compartments (RCs) where viral DNA (vDNA) is synthesized. While the recruitment of TLS polymerases is important for viral genome stability, the role of PCNA is poorly understood. PCNA function in DNA repair is regulated by monoubiquitination (mUb) or SUMOylation of PCNA at lysine 164 (K164). We find that mUb-PCNA increases over the course of infection, and modification of K164 is required for PCNA-mediated restriction of virus replication. mUb-PCNA plays important known roles in recruiting TLS polymerases to DNA, which we have shown are important for viral genome integrity and diversity, represented by structural variants and single nucleotide variants (SNVs), respectively. We find that PCNA drives SNVs on vDNA similar to Y-family TLS polymerases, but this did not require modification at K164. Unlike TLS polymerases, depeletion of PCNA did not result in large-scale rearrangements on vDNA. These striking results suggest separable PCNA-dependent and -independent functions of TLS polymerases on vDNA. By extension, these results imply roles for TLS polymerase beyond their canonical function in TLS in host biology. These findings highlight PCNA as a complex restriction factor for HCMV infection, likely with multiple distinct roles, and provide new insights into the PCNA-mediated regulation of DNA synthesis and repair in viral infection.

**IMPORTANCE:**

Genome synthesis is a critical step of virus life cycles and a major target of antiviral drugs. Human cytomegalovirus (HCMV), like other herpesviruses, encodes machinery sufficient for viral DNA synthesis and relies on host factors for efficient replication. We have shown that host DNA repair factors play important roles in HCMV replication, but our understanding of this is incomplete. Building on previous findings that specialized host DNA polymerases contribute to HCMV genome integrity and diversity, we sought to determine the importance of proliferating cell nuclear antigen (PCNA), the central polymerase regulator. PCNA is associated with nascent viral DNA and restricts HCMV replication. While PCNA is dispensable for genome integrity, it contributes to genome diversity. Our findings suggest that host polymerases function on viral genomes by separable PCNA-dependent and -independent mechanisms. Through revealing complex roles for PCNA in HCMV replication, this study expands the repertoire of host DNA synthesis and repair proteins hijacked by this ubiquitous herpesvirus.

## INTRODUCTION

Human cytomegalovirus (HCMV) is a ubiquitous herpesvirus that has co-evolved with humans and persists in a majority of the world’s population through the establishment of latent infection. Latency is defined as a quiescent infection where viral genomes are maintained in the absence of viral genome synthesis and viral progeny production (1). For HCMV, progenitor cells of the myeloid lineage and monocytes have been described as a major reservoir for latency (2, 3). While most seropositive individuals experience asymptomatic infection, HCMV reactivation from latency poses serious disease and mortality risk for immunocompromised individuals, including solid organ and stem cell transplant recipients. Infection during pregnancy and viral transmission to the fetus occurs in 1 in 200 births in the United States. Of these newborns with congenital infection, 20% will develop permanent disabilities, making HCMV the leading cause of infectious disease-related birth defects in the United States (4, 5). There are no clinical strategies to target or control latent HCMV infection due to limited knowledge of how the virus toggles between latent and replicative states. The complex interplay between viral determinants and host biology is central to the regulation of viral gene expression, genome amplification, and the decision to establish latency or replicate (6). Consequently, investigating these virus-host interactions at a molecular level is important to building a mechanistic understanding of latency and reactivation.

While HCMV encodes several proteins sufficient for viral DNA (vDNA) synthesis, it also relies on its host for specific factors that may fine-tune or modulate specific aspects of synthesis. HCMV infection drastically alters cell cycle progression (7) and induces E2F1 transcription factor activity, which regulates many host DNA synthesis and repair genes (8). Virus replication also activates DNA damage response signaling through major kinases, ataxia-telangiectasia mutated, and ataxia-telangiectasia Rad3 related (9). Many downstream repair proteins are re-localized to nuclear viral replication compartments (RCs), sites of vDNA synthesis, but their specific contributions to the viral replication cycle are poorly understood (8–11). In previous studies, we found that HCMV recruits specialized host DNA polymerases involved in translesion synthesis (TLS) pathways to modulate virus replication despite encoding its own DNA polymerase, *UL54*. TLS is a DNA damage bypass pathway in which specialized polymerases are recruited to replication forks or sites of damage in order to synthesize through lesions and prevent replication fork stalling or collapse (12). TLS polymerases lack proofreading activity and have a warped active site compared to replicative DNA polymerases, which allow for synthesis across DNA lesions albeit with lower fidelity (13). Therefore, TLS is a double-edged sword in which bulky nucleotide lesions may be bypassed during replication but at the cost of potentially introducing small point mutations into newly synthesized DNA. We found that the Y-family polymerases eta (η), kappa (κ), and iota (ι) restrict vDNA synthesis and viral replication, while the B-family polymerase zeta (ζ) and its putative scaffold, Y-family polymerase Rev1, are required for efficient vDNA synthesis and virus replication. Despite the opposing effects of these polymerases, all are required for viral genome integrity, whereby the depletion of either group of polymerases results in a significant increase in aberrant recombination and structural variants, primarily inversions, across the viral genome (14).

Proliferating cell nuclear antigen (PCNA) is an essential sliding clamp that acts as a processivity factor, maintaining polymerase association with DNA and promoting the processivity of DNA synthesis (15). Normally, PCNA interacts with B-family DNA polymerases delta (δ) and epsilon (ε) through a PCNA-interacting protein (PIP) motif (15). At sites of damage, PCNA is monoubiquitinated (mUb-PCNA) at lysine 164 (K164) by the E3 ubiquitin ligase, RAD18, to promote its association with TLS polymerases and subsequent damage bypass (16). TLS polymerases have less well-defined function in other DNA repair pathways that occur independently of PCNA (17–20). HCMV also encodes a viral DNA polymerase processivity factor, *UL44*, which is essential for virus replication (21). Like PCNA, pUL44 interacts with pUL54 to maintain polymerase-DNA interactions. While both PCNA and pUL44 are sliding clamps that associate with DNA, they share no sequence similarity (22). Additionally, PCNA is a homotrimer that undergoes extensive post-translational modifications to regulate interacting partners (23). By contrast, pUL44 is a homodimer with few characterized post-translational modifications and viral interacting partners (24–26). Despite these differences, pUL44 and pUL54 may function similarly to PCNA and cellular B-family polymerases as a complex to facilitate vDNA synthesis (21, 27). We sought to better understand the role of PCNA in HCMV replication.

We previously observed that HCMV infection in fibroblasts induces monoubiquitination of PCNA and, in line with observations from others, re-localization of PCNA to viral RCs (14, 28–30). This observation was consistent with the roles we found for TLS polymerases in regulating HCMV genome integrity and replication. However, the significance of PCNA to HCMV genome synthesis, integrity, and replication remains to be defined. Here, we found that PCNA restricted viral replication in the TB40/E strain in a manner that was dependent on its modification at K164. Homing in on this, we found that the accumulation of mUb-PCNA depended on viral DNA synthesis. Furthermore, PCNA and mUb-PCNA localized to distinct subdomains in RCs relative to replication forks and viral proteins important for vDNA synthesis. However, unlike the TLS polymerases that mUb-PCNA would presumably recruit, PCNA and mUb-PCNA were not required to protect vDNA from large rearrangements. Instead, we found that PCNA contributed to genome diversity through generating single nucleotide variants (SNVs) on vDNA, similar to Y-family TLS polymerases. These results suggest that, while the SNVs generated on vDNA by TLS polymerases are PCNA-dependent, TLS polymerase-moderated genome stability occurs independently of PCNA and likely post-synthesis. Altogether, this work uncovers specific contributions of PCNA to HCMV infection and highlights the complexity of this virus-host interaction.

## RESULTS

### mUb-PCNA increases with viral DNA synthesis

**Fig 1:**
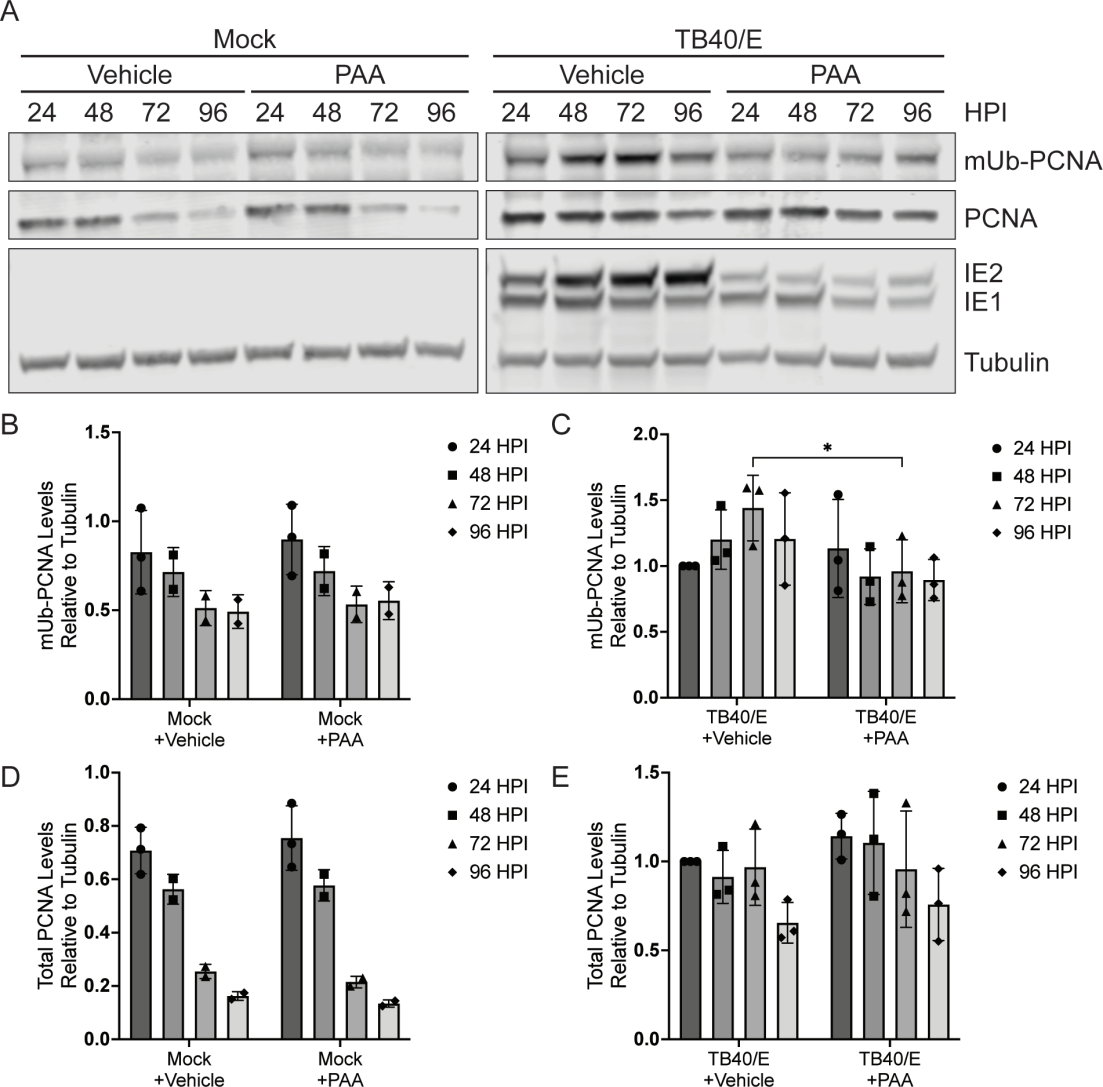
mUb-PCNA increases with vDNA synthesis. Fibroblasts were mock-infected or infected (multiplicity of infection [MOI] = 1) with TB40/E-WT virus over a 96-hour time course. Immunoblotting was performed on whole-cell lysates collected at the indicated time points. (A) mUb-PCNA and PCNA were detected using monoclonal antibodies specific to each and secondary antibodies conjugated to DyLight 680 (mouse) or 800 (rabbit). IE1/2 is an immediate early protein that serves as a marker for the progression of virus replication. Tubulin serves as a loading control. mUb-PCNA levels relative to tubulin were quantified in (B) mock-infected and (C) TB40/E-infected conditions and normalized to the 24 hpi time point. Total PCNA levels relative to tubulin were quantified in (D) mock-infected and (E) TB40/E-infected conditions and normalized to the infected 24 hpi time point. Statistical analysis was performed using two-way analysis of variance (ANOVA) with Tukey’s multiple comparisons test. Asterisks (*P < 0.05) represent statistically significant differences determined in three independent experiments.

Given the role of TLS polymerases in HCMV infection and their dependence on mUb-PCNA in host cells, we sought to build on our findings by further characterizing mUb-PCNA in the context of HCMV infection. To better define the relationships between PCNA, mUb-PCNA, and vDNA synthesis, we inhibited viral DNA synthesis with phosphonoacetic acid (PAA) and analyzed the accumulation of PCNA and mUb-PCNA. PAA is a small molecule that binds the viral polymerase, pUL54, and blocks the pyrophosphate binding site, ultimately inhibiting polymerase activity (31, 32). Cells were treated with PAA at the onset of infection (TB40/E-WT or mock). In mock-infected cells, mUb-PCNA decreased as cells grew to confluence, and PAA treatment had no effect on this over the 96 hours post-infection (hpi) time course (Fig. 1A, quantified in 1B). HCMV-infected cells accumulated mUb-PCNA as infection progressed, consistent with previous findings (14), while levels did not change in PAA-treated cells (Fig. 1A, quantified in 1C). Furthermore, unlike mock-infected cells, HCMV infection maintained mUb-PCNA despite increasing cell confluence. Total PCNA levels decreased in mock-infected cells as cells grew to confluence (Fig. 1D), while total levels in HCMV-infected cells were maintained but decreased moderately at late times of infection (Fig. 1E), consistent with previous findings (14). These data suggest that HCMV vDNA synthesis drives the accumulation of mUb-PCNA.

### PCNA restricts HCMV TB40/E replication

**Fig 2:**
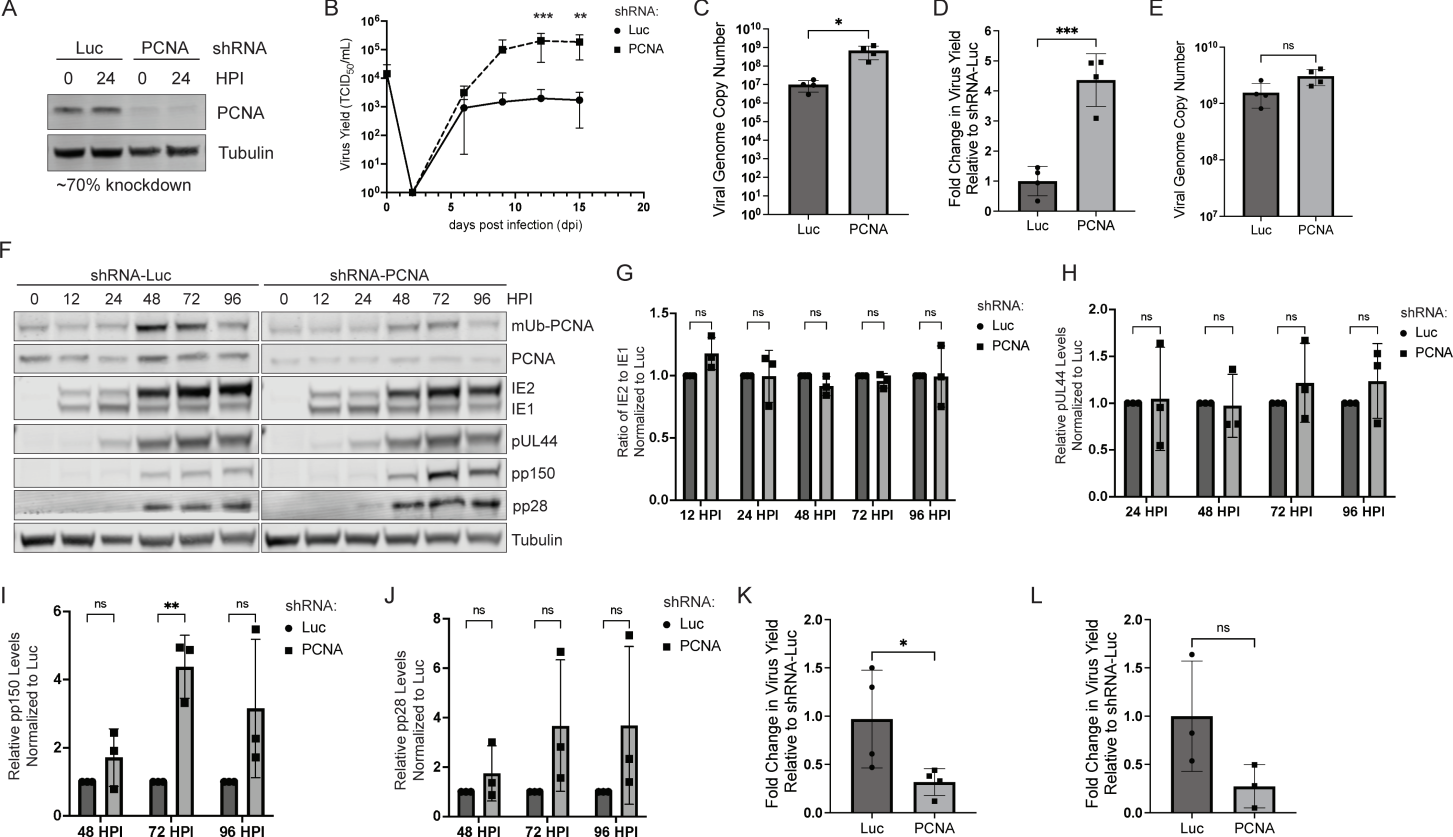
PCNA restricts HCMV TB40/E replication. (A–C) Growth-arrested fibroblasts expressing shRNA against PCNA or luciferase (Luc; non-targeting control) were infected with TB40/E-WT at an MOI of 0.02. (A) Whole-cell lysates were collected at the time of infection (0 hpi) and at 24 hpi and then immunoblotted. To confirm knockdown, PCNA was detected using a monoclonal antibody. An average knockdown of 70% was achieved over multiple independent experiments. (B) Virus yield at 15 dpi was determined by TCID_50_ and normalized relative to the Luc control. (C) Viral genome copy number at 15 dpi was determined by quantitative PCR (qPCR) using a TB40/E bacterial artificial chromosome (BAC) standard curve and primer set designed for the region of the viral genome encoding the b2.7 transcript. (D–F) Growth-arrested fibroblasts expressing shRNA against PCNA or Luc were infected with TB40/E-WT at an MOI of 1. (D) Virus yield at 96 hpi was determined by TCID_50_ and normalized relative to the Luc control. (E) The absolute viral genome copy number was determined by qPCR using a standard curve. (F) Whole-cell lysates were collected over a 96-hour time course (0 hpi = time of infection) and immunoblotted. To confirm knockdown, PCNA and mUb-PCNA were detected using monoclonal antibodies to each. The following proteins were also detected as markers for the viral gene expression cascade: IE1/2 (immediate early), pUL44 (early), and pp150 and pp28 (late) with secondary antibodies conjugated to DyLight 680 (mouse) or 800 (rabbit). Tubulin serves as a loading control. (G–J) Quantification of immunoblots in *F* for (G) IE1/2, (H) pUL44, (I) pp150, and (J) pp28 with PCNA knockdown normalized to Luc for each time point. (K) Growth-arrested fibroblasts expressing shRNAs were infected with HSV-1 at an MOI of 0.01, and virus yields were determined relative to the Luc control at 33 hpi. (L) Growth-arrested fibroblasts expressing shRNAs were infected with HCMV AD169-GFP (WT) at an MOI of 1, and virus yield was determined relative to the Luc condition at 96 hpi. For statistical analysis, significance was determined by two-way ANOVA with Tukey’s multiple comparisons test (B), two-way ANOVA with Sidak’s multiple comparisons test (G–J) or an unpaired *t* test (C–E and K–L). Asterisks (*P < 0.05, **P < 0.01, and ***P < 0.001) represent statistically significant differences determined in a minimum of three independent experiments.

Given that mUb-PCNA is associated with viral DNA synthesis, we hypothesized that PCNA is functionally important for viral replication. We stably disrupted PCNA expression via short hairpin RNA (shRNA) depletion under growth arrest conditions to avoid replication stress and cell death due to the depletion of this essential host factor. Compared to cells expressing shRNA against firefly luciferase (Luc, non-targeting control), we achieved ~70% knockdown of PCNA protein over multiple independent experiments (Fig. 2A). Compared to the Luc control, depletion of PCNA resulted in a ~2 log increase in virus yield, suggesting that PCNA restricts HCMV replication (Fig. 2B). Consistent with this, we also measured viral genome copy number at 15 days post-infection and observed an increase with depletion of PCNA (Fig. 2C). Cells depleted of PCNA infected at a multiplicity of infection (MOI) of 1 and collected over a 96-hour time course exhibited a fourfold increase in viral titers (Fig. 2D) but no significant increase in viral genomes (Fig. 2E). Therefore, PCNA restricts HCMV replication and genome synthesis, and the PCNA-mediated restriction of viral genome synthesis and progeny production was less apparent at higher MOIs of infection but not fully overcome.

We further analyzed the impact of PCNA on viral gene expression (Fig. 2F). Comparing Luc and PCNA knockdown, we observed no differences in immediate early proteins (IE1/2) or a representative early protein, pUL44. Protein levels of IE1/2 and pUL44 from three independent experiments are quantified in Fig. 2G and H, respectively. Therefore, PCNA does not impact viral gene expression early during infection. However, the late protein, pp150, was increased at 72 hpi, although increases in another late protein, pp28, did not reach statistical significance relative to the Luc control (Fig. 2I through J). Taken together, these data suggest that the restriction imposed by PCNA on vDNA synthesis is reflected in reduced viral gene expression late in infection.

Our observation that PCNA restricts HCMV replication is surprising considering that PCNA is re-localized to HCMV RCs and supports vDNA synthesis for other herpesviruses (33–35). Specifically, PCNA is required for replication of the alpha-herpesvirus, herpes simplex virus 1 (HSV-1) (35, 36), a finding that we recapitulate when we infect PCNA-depleted cells with HSV-1 (Fig. 2K). Furthermore, PCNA depletion has been shown to decrease genome synthesis in the lab-adapted AD169 strain of HCMV (30), which we also observe when measuring virus yield (Fig. 2L). These results suggest that the restriction imposed by PCNA on HCMV replication is due to genes or attributes specific to low-passage strains of HCMV.

### K164 modification on PCNA mediates restriction of HCMV TB40/E replication

To build upon our finding that PCNA restricts HCMV TB40/E replication, we sought to determine the significance of mUb-PCNA during infection. In response to DNA damage, PCNA is monoubiquitinated at K164 by RAD18, facilitating interactions with TLS polymerases (37, 38). PCNA is also polyubiquitinated at K164 to activate an alternate, error-free DNA lesion bypass through a post-replicative, template-switching mechanism (37, 39, 40). Furthermore, K164 may also be modified by small ubiquitin-like modification (SUMOylation) to antagonize homologous recombination DNA repair (41, 42). In order to investigate the significance of post-translational modifications on the K164 residue, we generated two shRNA-resistant PCNA constructs through wobble codon mutagenesis: wild-type PCNA and a mutant in which K164 is mutated to arginine (K164R), preventing both ubiquitination and SUMOylation on this residue.

**Fig 3:**
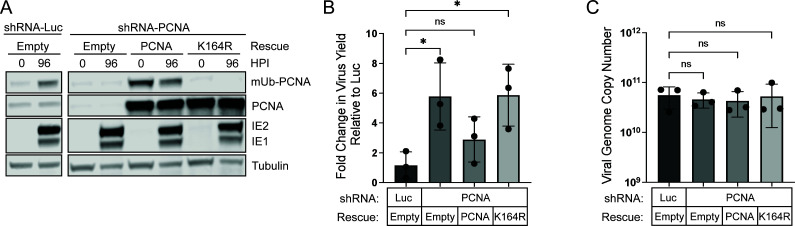
PCNA K164 modification mediates restriction of HCMV replication. Growth-arrested fibroblasts were transduced with lentiviral particles expressing shRNAs (targeting PCNA or luciferase). Simultaneously, cells were transduced to overexpress shRNA-resistant PCNA (wild-type or mutant containing a lysine-to-arginine mutation at amino acid 164 [K164R]) or an empty overexpression vector control. Cells were infected with HCMV at an MOI of 1 at 48 hours post-transduction and collected at 96 hpi. (A) To confirm protein knockdown and rescue, whole-cell lysates were collected at the time of infection (0 hpi) or at 96 hpi. mUb-PCNA, PCNA, tubulin, and IE1/2 were detected using monoclonal antibodies as shown with secondary antibodies conjugated to DyLight 680 (mouse) or 800 (rabbit). (B) Virus yields were measured by TCID_50_ and normalized relative to Luc. (C) Viral genome copy number was determined by qPCR using a TB40/E BAC standard curve and primer set designed for the region of the viral genome encoding the b2.7 transcript. Statistical significance was determined by one-way ANOVA with Tukey’s multiple comparisons test. Asterisks (**P < 0.01) represent statistically significant differences determined in independent experiments.

To determine how K164 modification on PCNA impacts HCMV replication, we generated lentivirus particles to deliver these constructs alongside shRNA in an attempt to rescue phenotypes associated with PCNA depletion. While overexpression of wild-type PCNA resulted in mUb-PCNA, expression of the K164R variant resulted in minimal detection of mUb-PCNA as expected (Fig. 3A). Cells expressing these constructs were infected and collected at 96 hpi. For controls, we also compared cells expressing shRNA against Luc or PCNA with no rescue (Empty).

As expected, PCNA knockdown with empty rescue recapitulated an approximately fivefold increase in virus yield over Luc control conditions. In comparison, PCNA knockdown with wild-type PCNA expression yielded no statistically significant change in virus replication compared to the Luc control, demonstrating a partial phenotypic rescue (Fig. 3B). Despite our findings that PCNA restricts HCMV replication, this partial rescue could be explained by differences in localization of the overexpressed protein, specifically its recruitment to viral RCs. PCNA-K164R expression resulted in replication at levels similar to PCNA depletion alone, suggesting that PCNA-mediated restriction of HCMV replication depends on modification of K164. Despite the effect on virus replication, PCNA-K164R did not impact viral genome copy number (Fig. 3C), similar to our results with PCNA knockdown at this MOI (Fig. 2E). Therefore, PCNA restricts HCMV replication in a manner dependent on modification at K164, although we cannot differentiate the importance of ubiquitination or SUMOylation of this residue.

### mUb-PCNA localizes to distinct replication compartment subdomains

PCNA localizes to viral RCs (14, 28–30), suggesting a role for PCNA in vDNA synthesis. However, the virus encodes its own functional homologue of PCNA, pUL44, that is important for processivity of the viral DNA polymerase, pUL54 (43, 44). To better understand the interplay between pUL44 and either PCNA or mUb-PCNA, we analyzed their localization in viral RCs and colocalization to replication forks. Active sites of vDNA synthesis labeled with 5-ethynyl-2′-deoxyuridine (EdU) have been localized to the periphery of RCs with pUL44 in HCMV infection (45), suggesting that viral DNA synthesis occurs at the periphery. However, others have observed active DNA synthesis throughout HCMV and HSV-1 RCs using either EdU or 5-ethynyl-2′-deoxycytidine nucleotide analogs (36, 46, 47). We used this technique to analyze the association of PCNA and mUb-PCNA with sites of active synthesis of vDNA relative to pUL44 or the HCMV single-stranded DNA binding protein, pUL57, which also localizes to RCs (45). To ensure labeling only of viral RCs, fibroblasts were growth arrested prior to infection and maintained in a serum-free conditions. At 48 hpi, we pulsed cells with EdU for 10 minutes and localized PCNA, mUb-PCNA, pUL44, and pUL57 by immunofluorescence to assess their association with EdU-labeled replication forks.

**Fig 4:**
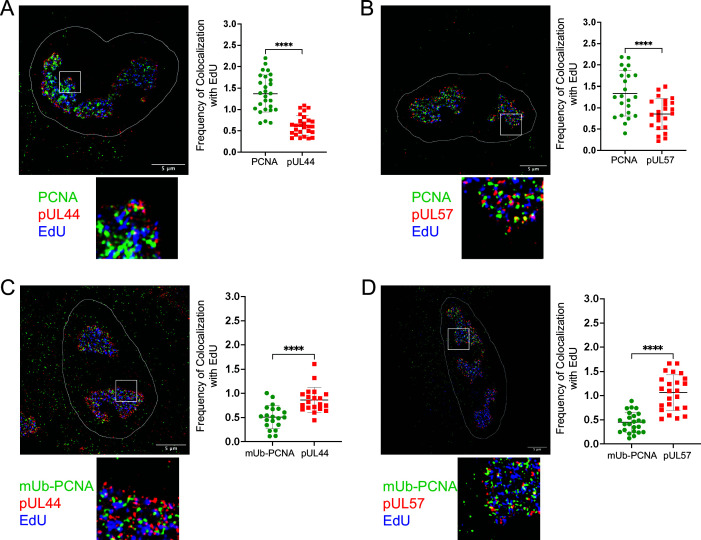
mUb-PCNA localizes to distinct RC subdomains. Fibroblasts were serum-starved and then infected with TB40/E-WT at an MOI of 1. At 48 hpi, cells were pulsed with 10 µM EdU for 10 minutes, cytoskeletal (CSK) extracted, and fixed. All coverslips were washed, and then a click reaction was performed to conjugate EdU and Alexa Fluor 647 (blue) for detection. Indirect immunofluorescence was then carried out using monoclonal antibodies to the indicated proteins: (A) PCNA and pUL44, (B) PCNA and pUL57, (C) mUb-PCNA and pUL44, and (D) mUb-PCNA and pUL57 with secondary antibodies conjugated to Alexa Fluor 546 (green) or 488 (red). 4′,6-Diamidino-2-phenylindole (DAPI)-stained nuclei (not pictured) were outlined using Fiji/ImageJ software. An enlargement of the boxed area is shown below each image. Images were captured using a Zeiss Elyra S.1 super-resolution microscope. Scale bar, 5 µm. The frequency of colocalization between host or viral proteins and EdU was quantified using Nikon NIS Elements software (see Materials and Methods) and is shown to the right of each image with each point representing a cell. Statistical significance was determined by a paired *t* test. Asterisks (****P < 0.0001) represent statistically significant differences determined in three independent experiments.

EdU labeling was specifically incorporated throughout the RCs of infected cells (Fig. 4). PCNA more frequently colocalized with EdU than either pUL44 (Fig. 4A) or pUL57 (Fig. 4B), suggesting a role at or near the replication fork. However, the association of mUb-PCNA with EdU was less than that of pUL44 (Fig. 4C) or pUL57 (Fig. 4D), suggesting a role more distant from the replication fork and possibly occuring post-synthesis. Because we did not observe EdU incorporation at the periphery of RCs as previously reported by Strang et al. (45), we analyzed EdU labeling in cells infected with the AD169 laboratory-adapted strain in case virus strain accounted for these differences. EdU was incorporated throughout the RCs similarly to TB40/E infection (Fig. S1). The differences between the observations reported here and those by Strang et al. may be due to cell type or MOI differences. However, incorporation of EdU throughout RCs has been reported by others in HCMV and HSV-1 infection (36, 46, 47).

### PCNA contributes to HCMV genome diversity but not integrity

**Fig 5:**
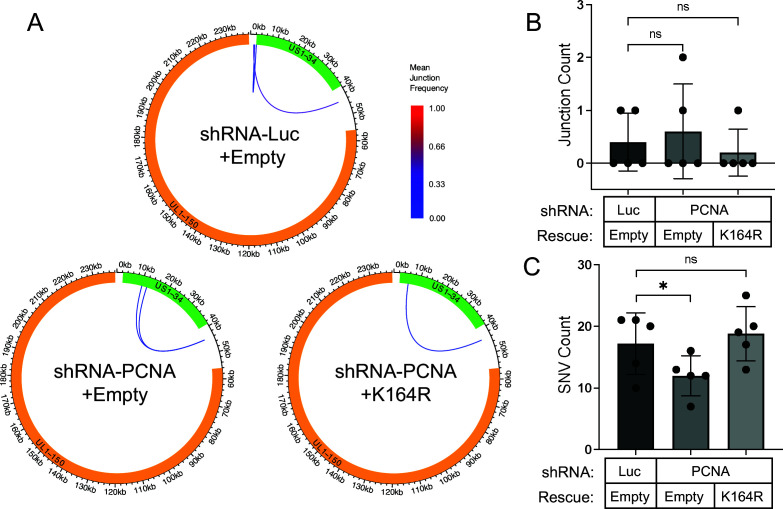
PCNA contributes to HCMV genome diversity but not integrity. Growth-arrested fibroblasts were transduced with lentiviral particles expressing shRNAs against PCNA or luciferase at an MOI of 3. Simultaneously, cells were transduced to overexpress shRNA-resistant PCNA-K164R or an empty overexpression vector control. Forty-eight hours later, media were refreshed with puromycin at 2 µg/mL; 24 hours later, cells were infected with TB40/E-WT at an MOI of 1, and total DNA was isolated at 96 hpi for sequencing. Sequences from each knockdown condition as well as from the virus stock used for infection were aligned to the TB40/E-GFP reference genome. (A) Mean novel junction frequency within each condition. HCMV genomic coordinates are plotted along the circular axis in graphs for each condition, and the UL (orange) and US (green) regions of the genome are marked. The arcs connect novel junction points detected at the average frequency for the given condition indicated by the color scale. (B) Quantification of the number of novel junctions (inversions, deletions, and duplications) detected per sample (*n* = 5) for each condition. (C) Quantification of the number of novel SNVs (point mutations, deletions, or insertions) detected per sample (*n* = 5) for each condition. Statistical significance was determined by pairwise two-sided exact Poisson tests and adjusted using Bonferroni correction. Asterisks (*P < 0.05) represent statistically significant differences determined in independent experiments.

Our findings thus far point to a role for PCNA in regulating HCMV genome synthesis and replication. As an essential factor for eukaryotic DNA replication and repair, we wondered about the extent to which PCNA influences viral genome integrity. In previous studies, we found that TLS polymerases are required for HCMV genome integrity (14). While TLS polymerases can function in DNA repair independently of PCNA (17, 19, 20), mUb-PCNA is critical to recruit TLS polymerases to lesions for canonical translesion synthesis (15, 23). Building on the findings presented here, we sought to determine if depletion of PCNA alone was sufficient to compromise viral genome integrity in HCMV TB40/E infection and if genome integrity depended on modification at K164. To assess this, we sequenced genomic DNA extracted from HCMV-infected fibroblasts expressing shRNAs against luciferase or PCNA with expression of an empty vector or PCNA-K164R as described (Fig. 3A) and quantitated novel DNA junctions (inversions, duplications, and deletions) and SNVs (point mutations, small deletions, and insertions) arising in synthesized viral genomes compared to the parental virus stock used for infection. Strikingly, we found that neither depletion of PCNA nor rescue with the K164R mutant significantly impacted large genomic rearrangements compared to the Luc control (Fig. 5A, quantified in 5B). This was a surprising result given the role of PCNA in recruiting TLS polymerases. By contrast, the depletion of TLS polymerases increased inversions, duplications, and deletions across the viral genome (14). However, similar to the depletion of TLS polymerases (14), the depletion of PCNA resulted in fewer SNVs on the viral genome, but SNVs were generated independently of modification at K164 (Fig. 5C). Taken together, these results suggest that PCNA is dispensable in the maintenance of HCMV genome integrity but could drive genome diversity.

## DISCUSSION

**Fig 6:**
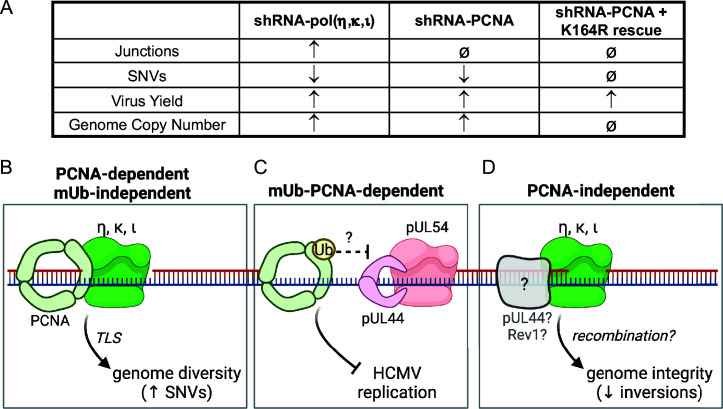
Model for the varied roles of PCNA and TLS polymerases in HCMV DNA synthesis and genome integrity. (A) Summary of observed virus infection phenotypes with host factor knockdown. The table depicts an observed increase (↑), decrease (↓), or no change (ø) in phenotypes listed for each unique condition relative to control (shRNA-Luc). (B) PCNA and TLS polymerases are required for viral genome diversity. In a mUb-PCNA-independent manner, PCNA and error-prone Y-family TLS polymerases η, κ, and ι engage in TLS repair on vDNA, contributing to the generation of SNVs and genome diversity. This may occur at replication forks by canonical TLS bypass. (C) PCNA restricts HCMV replication dependent on its modification at K164. While an exact mechanism remains to be defined, PCNA could compete with or inhibit functions of the viral processivity factor, pUL44, and DNA polymerase, pUL54. These activities may occur post-synthesis rather than at replication forks. (D) TLS polymerases have PCNA-independent functions in HCMV genome integrity. Y-family TLS polymerases η, κ, and ι maintain HCMV genome integrity by preventing inversions on vDNA, likely through recombination-dependent repair. This repair is independent of PCNA and possibly involves an alternative factor, such as pUL44 or Rev1. Created in BioRender (F. Goodrum, 2025, https://BioRender.com/x06k376).

As intracellular parasites, all viruses rely on host cell factors for efficient replication. Even complex DNA viruses, like herpesviruses, hijack the host’s machinery despite encoding similar factors of their own. Our current knowledge of the involvement of host factors in the HCMV replicative program, especially at the step of vDNA synthesis, is incomplete. Building on our previous findings that TLS polymerases are recruited to viral RCs and modulate HCMV replication and genome integrity (14), we set out to define the roles of the host DNA processivity factor and polymerase-binding partner, PCNA, in HCMV replication. While PCNA is best known for its role in increasing polymerase processivity, it also plays an important role in DNA repair (15). Post-translational modifications, such as ubiquitination or SUMOylation, direct its engagement with repair pathways. We show that HCMV infection induces monoubiquitination of PCNA, a modification important for recruiting TLS polymerases. PCNA activity on vDNA restricts vDNA synthesis and HCMV replication, and post-translational modifications (i.e., monoubiquitination or SUMOylation) at the K164 residue are important for this restriction (Fig. 3B). Despite having a role in viral DNA synthesis, PCNA depletion did not affect viral genome integrity (Fig. 5A and B), as depletion of TLS polymerases does (14). However, PCNA contributed to SNVs on viral DNA, similar to Y-family TLS polymerases, η, κ, and ι (Fig. 5C, summarized in 6A), suggesting that TLS polymerases function on the viral genome by both PCNA-dependent and -independent mechanisms. Additionally, unmodified PCNA and mUb-PCNA were differentially associated with sites of active vDNA synthesis (Fig. 4), further emphasizing the multifaceted role of PCNA in regulating HCMV replication. This work highlights PCNA as a restriction factor to HCMV replication with complex roles that remain to be defined, as summarized in our proposed model (Fig. 6).

With no enzymatic activity, PCNA undergoes multiple post-translational modifications to direct chromatin binding and association with interacting partners. Of these modifications, monoubiquitination by the E3 ubiquitin ligase, RAD18, is important for the recruitment of specialized polymerases to bypass obstructive DNA lesions on cellular DNA (16, 38). Our observation that induction of mUb-PCNA is associated with vDNA synthesis (Fig. 1) suggests this is a virus-driven phenomenon. However, the induction of mUb-PCNA may also be a host response to virus infection. For example, increased virus replication is associated with increased oxidative stress (48, 49), and mUb-PCNA induction has been associated with oxidative stress (50, 51). Additionally, the induction of mUb-PCNA and its localization to viral RCs could be a response to replication stress that arises due to vDNA synthesis in the nucleus. In this scenario, the association of PCNA with viral genomes might interfere with vDNA synthesis machinery (i.e., pUL54 and pUL44) and ultimately cause a restriction to vDNA synthesis (Fig. 6C). To further understand the nature and significance of PCNA-K164, future studies should examine the host and/or viral proteins that interact with PCNA and regulate post-translational modifications to direct PCNA function during infection.

We report that PCNA restricts HCMV-TB40/E replication based on observations that shRNA knockdown of PCNA resulted in increased virus yield (Fig. 2). This was a surprising finding considering that HCMV induces PCNA expression (52, 53), and PCNA localizes to RCs (28, 54) with newly synthesized viral DNA (Fig. 5). Furthermore, PCNA facilitates the replication of other herpesviruses, HSV-1 (35) and Epstein Barr virus (EBV) (55). For HSV-1, PCNA plays a key role by recruiting viral and cellular factors to sites that facilitate viral genome synthesis (36). Various distinctions between HSV-1 and HCMV, including genome size and differences in the viral processivity factors and kinetics of replication, may underlie how these viruses differentially utilize PCNA. Furthermore, our findings with the low-passage TB40/E strain stand in contrast to previous findings showing that PCNA knockdown decreased HCMV viral genome production in studies using the AD169 strain (30). While we observe that PCNA restricts viral genome synthesis in low MOI infections of TB40/E (Fig. 2C), it is possible that strain differences account for the discrepancy in results. In our study, PCNA knockdown in AD169 infection did not produce a significant change in virus replication (Fig. 2L). AD169 notably lacks 15 kb of viral DNA including many genes important for modulating viral replication for establishment of latency (56). Thus, it is conceivable that some of these genes modulate PCNA in a way that restricts virus replication. For example, pUL145 has been reported to degrade helicase-like transcription factor (57), a multi-functional protein that contains a RING domain that interacts with RAD18 to promote polyubiquitination on PCNA-K164 (40, 58). Additionally, we recently defined an interaction between the *ULb’* gene product, UL138, and host deubiquitinating enzyme (DUB), ubiquitin-specific protease 1 (USP1), an important regulator of mUb-PCNA and the TLS pathway (59). As both UL138 and USP1 restrict HCMV replication, it is possible that this interaction influences the effect of PCNA on virus replication. Therefore, future studies investigating the regulation of PCNA by *ULb’* genes may provide insight into strain-dependent differences.

While the mechanisms by which PCNA restricts HCMV infection remain to be defined, we report that K164 modification is important to this restriction in TB40/E infection. Depletion of PCNA with overexpression of wild-type PCNA yielded no significant change compared to control conditions, but overexpression of the K164R mutant replicated significantly more than the control (Fig. 3). We speculate that the partial phenotypic rescue with wild-type PCNA could be due to differences in localization of the overexpressed protein, and viral RCs recruit specific levels of PCNA. Consequently, the function of PCNA, determined by its ability to undergo post-translational modification, within RCs would be a key factor in modulating virus replication. Multiple studies have demonstrated that regulation of PCNA and its post-translational modifications are important for other herpesvirus infections. During infection by the gamma-herpesvirus, EBV, PCNA is deubiquitinated by the viral enzyme, BPLF1 (60). Similarly, PCNA ubiquitination is induced during productive HSV-1 infection and antagonized by the viral DUB, UL36USP (61). Both of these studies showed that deubiquitination of PCNA was important for downregulating polη recruitment to sites of DNA damage outside the context of infection, but the role of PCNA ubiquitination during viral infection remains to be determined. Notably, Whitehurst et al. show that a PIP domain is conserved among herpesvirus-encoded DUBs, suggesting that these viral enzymes could regulate PCNA. HCMV pUL48 has DUB activity (62); however, a role for pUL48 or host DUBs in deubiquitinating PCNA during infection has yet to be defined. It is possible that herpesvirus infections commonly induce mUb-PCNA, but its significance to infection varies among different viruses.

PCNA monoubiquitination at K164 is thought to primarily coordinate the DNA damage bypass pathway, TLS. We previously found that TLS polymerases are involved in HCMV infection, whereby Y-family insertion polymerases η, κ, and ι restricted replication (Fig. 6A), and Rev1 and ζ were required for efficient virus replication (14). Like the insertion TLS polymerases, PCNA restricts HCMV replication, suggesting they could work together through canonical TLS to achieve this effect. Furthermore, similar to previous findings with depletion of TLS polymerases η, κ, and ι (14), we observed a moderate but statistically significant decrease in SNVs on vDNA with depletion of PCNA (Fig. 5C and 6A). These observations suggest that PCNA-regulated TLS occurs on the HCMV genome to drive SNVs, consistent with the error-prone nucleotide insertion function of these polymerases (17). Surprisingly, however, PCNA knockdown and expression of the K164R mutant did not impact SNV counts, suggesting that, while PCNA is important for the generation of SNVs, it occurs independently of the mUb-PCNA thought to recruit TLS polymerases to DNA (Fig. 6B).

In contrast to SNVs, PCNA depletion did not increase structural variants on the genome, indicating no requirement for HCMV genome integrity (Fig. 5A and B), while depletion of TLS polymerases results in increased novel junctions, primarily inversions (summarized in Fig. 6A) (14). Furthermore, rescue of PCNA knockdown with the K164R mutant failed to restore the PCNA-mediated restriction to viral replication (suggesting a requirement for modification at K164; Fig. 3) but had no effect on genome integrity (suggesting the modification on K164 is dispensable for maintaining integrity; Fig. 5). These data suggest that TLS polymerase regulation of HCMV genome integrity occurs independently of PCNA and its monoubiquitination (Fig. 6D). The fact that depletion of TLS polymerases increased genomic rearrangements, introducing novel junctions on vDNA primarily through sequence inversions (14), suggests that TLS polymerases are engaging in homology-directed repair, and this occurs independently of PCNA and occurs post-synthesis by non-canonical TLS functions given the distance of mUb-PCNA from the replication fork. The function of TLS polymerases in homology-directed recombination is poorly defined (17), and HCMV offers a new tool for defining these roles. For example, polη and polκ have been implicated in DNA repair at common fragile sites or difficult-to-replicate regions through a mUb-PCNA-independent mechanism (17, 18). Polη can also function in homology-directed repair pathways, such as break-induced replication, without the involvement of PCNA (19, 20). Intriguingly, the PCNA-independent functions of TLS polymerases are associated with recombination, suggesting that HCMV commandeers Y-family polymerases, but not PCNA, for recombination-dependent repair. This is an intriguing avenue for future studies. However, one possible limitation of these interpretations as they apply to the study is that because we only deplete PCNA levels by 70%, it is possible that the remaining protein is sufficient to permit repair in the presence of TLS polymerases. However, given the phenotypes resulting from the depletion of PCNA on vDNA synthesis, late gene expression, replication, and the generation of SNVs, we think this is unlikely.

Furthermore, it remains a possibility that in the absence of PCNA, TLS polymerases are recruited by pUL44 or an alternative factor, such as Rev1 (63–65). This would suggest that PCNA and pUL44 compete for interacting partners and provide a possible explanation as to why PCNA restricts HCMV replication. However, our localization studies (Fig. 4) show poor co-localization of PCNA and pUL44 at EdU-labeled replication forks, and PCNA is more frequently associated with replication forks than either pUL44 or pUL57. Future studies will address the roles of other factors, such as viral pUL44 or host Rev1, in TLS polymerase repair activity during infection.

mUb-PCNA is less frequently associated with EdU-labeled replication forks than pUL44 or pUL57, suggesting possible post-synthesis roles that are likely not related to TLS-mediated lesion bypass, (Fig. 4). The differential subnuclear localization of unmodified and mUb-PCNA implies specific, separable functions of PCNA. It is possible that mUb-PCNA is induced to prevent K164 modification by SUMOylation, which suppresses DNA repair by the homologous recombination pathway (41, 42). This would support data in Fig. 3 showing that PCNA’s suppressive effect on HCMV TB40/E replication is mediated by K164 modification. However, further investigation is required to attribute this to monoubiquitination, polyubiquitination, SUMOylation, or a combination of these modifications. Additionally, the ubiquitination of PCNA is linked to its retention on chromatin and regulation of nucleosome deposition (66), a function required for HSV-1 infection (35). As PCNA interacts with a wide array of repair proteins, further investigation is required to define mUb-PCNA-associated host and/or viral proteins to elucidate this function.

In summary, our findings underscore the intricacy of HCMV interactions with its host, especially with respect to DNA synthesis and repair. Through studying the role of PCNA in HCMV infection, we uncovered multiple, distinct functions of PCNA and, by extension, TLS polymerases on viral genomes that deviate from their canonical function in TLS in host cell biology (Fig. 6). While TLS polymerases engage in canonical TLS repair of vDNA, which is likely PCNA-dependent, but mUb-PCNA-independent (Fig. 6B), they also likely function in a PCNA-independent manner to protect viral DNA from faulty recombination (Fig. 6D). Additional work is required to understand specific mechanisms of host machinery-mediated repair of viral genomes by TLS polymerases and the role of mUb-PCNA in restricting virus replication (Fig. 6C). HCMV infects a large diversity of cell types and has a large, complex exogenous genome, which is readily manipulated. Furthermore, the vDNA is synthesized in the context of cell cycle arrest and inhibition of host DNA synthesis. This allows for the knockdown of critical host factors important to synthesis or repair that would otherwise result in stress or cell death, confounding any results for the requirement in host cell or infection biology. Therefore, HCMV offers an exciting tool to further mechanistically define and separate complex, intermingled DNA synthesis and repair pathways.

## MATERIALS AND METHODS

### Cells and viruses

Primary human lung MRC-5 fibroblasts (ATCC CCL-171) were maintained in Dulbecco's modified Eagle medium (DMEM) containing 10% fetal bovine serum (FBS) as previously described (67). Cells were infected with the low-passage HCMV strain, TB40/E, a gift from Dr. Christian Sinzger.

### RNA interference (RNAi)

Control shRNA targeting luciferase was purchased from Sigma-Aldrich (#SHC007). The shRNA targeting PCNA was constructed in the pLKO.1 backbone (14). In brief, a 21-mer oligonucleotide (GAATGAACCAGTTCAACTAAC) was generated for the target gene and then cloned into pLKO.1 vector via annealing.

### Lentivirus and transduction

Lentiviral particles for shRNA delivery were cultured in HEK 293T cells as previously described (14). For transduction, MRC-5 fibroblasts were grown to confluence and growth-arrested by contact inhibition to avoid the deleterious effects of PCNA depletion in cells undergoing division. Cells were transduced with lentivirus shRNA or rescue constructs at an MOI of 3 in media containing 1 µg/mL polybrene. At 2 days post-transduction, cells were washed in PBS and then given fresh growth media. At 4 days post-transduction, cells were infected with HCMV as described.

### Plasmids

The genetic sequence for human PCNA was amplified from pME-GFP-PCNA (Addgene #105977). Primers containing a sense mutation for shRNA target sequence were used to amplify shRNA-resistant PCNA. Primers containing K164R substitution were used on this sequence to amplify shRNA-resistant PCNA-K164R. PCNA and PCNA-K164R were then expressed from a pCIG vector. All plasmid sequences were validated by Sanger sequencing.

### EdU pulse labeling and immunofluorescence

Fibroblasts were seeded onto 1.5H high precision coverslips (Marienfeld Superior) in 24-well plates and maintained in serum-free culture medium. At 48 hpi, cells were pulsed with EdU for 10 minutes and then processed for indirect immunofluorescence (14). For detection of EdU, the click reaction was performed according to manufacturer instructions (Invitrogen #C10340) after the fixation step.

### Colocalization analysis of host and viral proteins to EdU staining in host cell nuclei

Nikon NIS Elements AR 5.42.03 software with the General Analysis 3 (GA3) module was used for image processing and analysis. An in-depth description of the protocol is provided in Supplemental Material.

### Immunoblotting

Whole-cell lysates were extracted using radioimmunoprecipitation assay (RIPA) lysis buffer (Pierce) and manual scraping. Fifty microgram of lysate was loaded onto precast 4%–12% bis-tris gels (ExpressPlus; GenScript), and then proteins were separated by electrophoresis and transferred onto 0.45 µm pore size polyvinylidene fluoride (PVDF) membranes (Immobilon-FL; Millipore). Proteins of interest were detected using primary and fluorophore-conjugated secondary antibodies (Table 1). Images were obtained using a Li-Cor Odyssey CLx scanner, and protein levels were quantitated using Image Studio Lite software.

**TABLE 1:** Antibodies used in this study

Antibody	Species	Source	Concentration
PCNA	Mouse	Santa Cruz, sc-56	IB 1:1,000
PCNA	Rabbit	Cell Signaling Technology (CST), #13110	IF 1:400
mUb-PCNA	Rabbit	CST, #13439	IB 1:1,000 IF 1:100
a-Tubulin	Mouse	Sigma-Aldrich, #T9026	IB 1:2,000
IE1/2	Mouse	Thomas Shenk, PhD (Princeton University)	IB 1:100
pUL44	Mouse	Virusys, #CA006	IB, IF 1:12,000
pUL57	Mouse	Virusys, #P1209	IF 1:100
pp150	Mouse	William J. Britt, MD (University of Alabama at Birmingham)	IB 1:15
pp28	Mouse	William J. Britt, MD (University of Alabama at Birmingham)	IB 1:15
Mouse IgG (H + L) secondary, DyLight 680	Goat	Invitrogen, #35519	IB: 1:6,000
Rabbit IgG (H + L) secondary, DyLight 800	Goat	Invitrogen, #SA5-10036	IB: 1:6,000
Mouse IgG (H + L) secondary, Alexa Fluor 488	Goat	Invitrogen, #A-11029	IF: 1:3,000
Rabbit IgG (H + L) secondary, Alexa Fluor 546	Goat	Invitrogen, #A-11035	IF 1:3,000
Mouse IgG (H + L) secondary, Alexa Fluor 647	Goat	Invitrogen, #A-21236	IF: 1:3,000

### Genomic sequencing and computational analysis

Fibroblasts were seeded onto 6 cm dishes and transduced with lentiviral constructs as described above. Five technical replicates were seeded for each condition. At 96 hpi, DNA was purified from infected cells as previously described (14). All purified DNA was submitted to SeqCenter (Pittsburgh, PA) and subsequently analyzed as previously described (14). An in-depth description is provided in Supplemental Material.

## Data Availability

Alignments used for Fig. 5 are available at the University of Arizona Research Data Repository (doi: 10.25422/azu.data.27948387). Raw sequence reads have been deposited in the Sequence Read Archive, https://www.ncbi.nlm.nih.gov/sra (BioProject ID: PRJNA1220172).

## SUPPLEMENTAL MATERIAL

Supplemental material mbio.00450-25-s0001.docx Fig. S1 and extended materials and methods.

ASM does not own the copyrights to Supplemental Material that may be linked to, or accessed through, an article. The authors have granted ASM a non-exclusive, world-wide license to publish the Supplemental Material files. Please contact the corresponding author directly for reuse.

## References

[1] Goodrum F. 2016. Human cytomegalovirus latency: approaching the gordian knot. Annu Rev Virol 3:333–357. doi:10.1146/annurev-virology-110615-042422 27501258 PMC5514425

[2] von Laer D, Meyer-Koenig U, Serr A, Finke J, Kanz L, Fauser A, Neumann- Haefelin D, Brugger W, Hufert F. 1995. Detection of cytomegalovirus DNA in CD34+ cells from blood and bone marrow. Blood 86:4086–4090. doi:10.1182/blood.V86.11.4086.bloodjournal86114086 7492764

[3] Mendelson M, Monard S, Sissons P, Sinclair J. 1996. Detection of endogenous human cytomegalovirus in CD34+ bone marrow progenitors. J Gen Virol 77 (Pt 12):3099–3102. doi:10.1099/0022-1317-77-12-3099 9000102

[4] Boppana SB, Ross SA, Fowler KB. 2013. Congenital cytomegalovirus infection: clinical outcome: prenatal therapy of congenital cytomegalovirus infection. Clin Infect Dis 57:S178–S181. doi:10.1093/cid/cit629 24257422 PMC4471438

[5] Cannon MJ. 2009. Congenital cytomegalovirus (CMV) epidemiology and awareness. J Clin Virol 46 Suppl 4:S6–10. doi:10.1016/j.jcv.2009.09.002 19800841

[6] Mlera L, Moy M, Maness K, Tran LN, Goodrum FD. 2020. The role of the human cytomegalovirus UL133-UL138 Gene Locus in Latency and Reactivation. Viruses 12:714. doi:10.3390/v12070714 32630219 PMC7411667

[7] Spector DH. 2015. Human cytomegalovirus riding the cell cycle. Med Microbiol Immunol 204:409–419. doi:10.1007/s00430-015-0396-z 25776080

[8] E X, Pickering MT, Debatis M, Castillo J, Lagadinos A, Wang S, Lu S, Kowalik TF. 2011. An E2F1-mediated DNA damage response contributes to the replication of human cytomegalovirus. PLoS Pathog 7:e1001342. doi:10.1371/journal.ppat.1001342 21589897 PMC3093362

[9] E X, Kowalik T. 2014. The DNA damage response induced by infection with human cytomegalovirus and other viruses. Viruses 6:2155–2185. doi:10.3390/v6052155 24859341 PMC4036536

[10] Luo MH, Rosenke K, Czornak K, Fortunato EA. 2011. Human cytomegalovirus disrupts both ataxia telangiectasia mutated protein (ATM)- and ATM-Rad3-related kinase-mediated DNA damage responses during lytic infection. J Virol 85:3043–3043. doi:10.1128/JVI.00008-11 PMC1797560 17151099

[11] O’Dowd JM, Zavala AG, Brown CJ, Mori T, Fortunato EA. 2012. HCMV-infected cells maintain efficient nucleotide excision repair of the viral genome while abrogating repair of the host genome. PLoS Pathog 8:e1003038. doi:10.1371/journal.ppat.1003038 23209410 PMC3510244

[12] Marians KJ, Kornberg RD. 2018. Lesion bypass and the reactivation of stalled replication forks. Annu Rev Biochem 87:217–238. doi:10.1146/annurev-biochem-062917-011921 29298091 PMC6419508

[13] Waters LS, Minesinger BK, Wiltrout ME, D’Souza S, Woodruff RV, Walker GC. 2009. Eukaryotic translesion polymerases and their roles and regulation in DNA damage tolerance. Microbiol Mol Biol Rev 73:134–154. doi:10.1128/MMBR.00034-08 19258535 PMC2650891

[14] Zeltzer S, Longmire P, Svoboda M, Bosco G, Goodrum F. 2022. Host translesion polymerases are required for viral genome integrity. Proc Natl Acad Sci USA 119. doi:10.1073/pnas.2203203119 PMC9388158 35947614

[15] Choe KN, Moldovan G-L. 2017. Forging ahead through darkness: PCNA, still the principal conductor at the replication fork. Mol Cell 65:380–392. doi:10.1016/j.molcel.2016.12.020 28157503 PMC5302417

[16] Yang W, Gao Y. 2018. Translesion and repair DNA polymerases: diverse structure and mechanism. Annu Rev Biochem 87:239–261. doi:10.1146/annurev-biochem-062917-012405 29494238 PMC6098713

[17] Paniagua I, Jacobs JJL. 2023. Freedom to err: the expanding cellular functions of translesion DNA polymerases. Mol Cell 83:3608–3621. doi:10.1016/j.molcel.2023.07.008 37625405

[18] Barnes RP, Hile SE, Lee MY, Eckert KA. 2017. DNA polymerases eta and kappa exchange with the polymerase delta holoenzyme to complete common fragile site synthesis. DNA Repair (Amst) 57:1–11. doi:10.1016/j.dnarep.2017.05.006 28605669 PMC9642814

[19] McIlwraith MJ, Vaisman A, Liu Y, Fanning E, Woodgate R, West SC. 2005. Human DNA polymerase eta promotes DNA synthesis from strand invasion intermediates of homologous recombination. Mol Cell 20:783–792. doi:10.1016/j.molcel.2005.10.001 16337601

[20] Buisson R, Niraj J, Pauty J, Maity R, Zhao W, Coulombe Y, Sung P, Masson J-Y. 2014. Breast cancer proteins PALB2 and BRCA2 stimulate polymerase η in recombination-associated DNA synthesis at blocked replication forks. Cell Rep 6:553–564. doi:10.1016/j.celrep.2014.01.009 24485656 PMC4162405

[21] Zarrouk K, Piret J, Boivin G. 2017. Herpesvirus DNA polymerases: structures, functions and inhibitors. Virus Res 234:177–192. doi:10.1016/j.virusres.2017.01.019 28153606

[22] Appleton BA, Brooks J, Loregian A, Filman DJ, Coen DM, Hogle JM. 2006. Crystal structure of the cytomegalovirus DNA polymerase subunit UL44 in complex with the C terminus from the catalytic subunit. Differences in structure and function relative to unliganded UL44. J Biol Chem 281:5224–5232. doi:10.1074/jbc.M506900200 16371349

[23] Ulrich HD. 2009. Regulating post-translational modifications of the eukaryotic replication clamp PCNA. DNA Repair (Amst) 8:461–469. doi:10.1016/j.dnarep.2009.01.006 19217833

[24] Sinigalia E, Alvisi G, Segré CV, Mercorelli B, Muratore G, Winkler M, Hsiao H-H, Urlaub H, Ripalti A, Chiocca S, Palù G, Loregian A. 2012. The human cytomegalovirus DNA polymerase processivity factor UL44 is modified by SUMO in a DNA-dependent manner. PLoS One 7:e49630. doi:10.1371/journal.pone.0049630 23166733 PMC3499415

[25] Strang BL, Sinigalia E, Silva LA, Coen DM, Loregian A. 2009. Analysis of the association of the human cytomegalovirus DNA polymerase subunit UL44 with the viral DNA replication factor UL84. J Virol 83:7581–7589. doi:10.1128/JVI.00663-09 19457994 PMC2708651

[26] Krosky PM, Baek M-C, Jahng WJ, Barrera I, Harvey RJ, Biron KK, Coen DM, Sethna PB. 2003. The human cytomegalovirus UL44 protein is a substrate for the UL97 protein kinase. J Virol 77:7720–7727. doi:10.1128/jvi.77.14.7720-7727.2003 12829811 PMC161957

[27] Loregian A, Appleton BA, Hogle JM, Coen DM. 2004. Residues of human cytomegalovirus DNA polymerase catalytic subunit UL54 that are necessary and sufficient for interaction with the accessory protein UL44. J Virol 78:158–167. doi:10.1128/jvi.78.1.158-167.2004 14671097 PMC303418

[28] Lee SB, Lee CF, Ou DSC, Dulal K, Chang LH, Ma CH, Huang CF, Zhu H, Lin YS, Juan LJ. 2011. Host-viral effects of chromatin assembly factor 1 interaction with HCMV IE2. Cell Res 21:1230–1247. doi:10.1038/cr.2011.53 21445097 PMC3193474

[29] Dittmer D, Mocarski ES. 1997. Human cytomegalovirus infection inhibits G1/S transition. J Virol 71:1629–1634. doi:10.1128/JVI.71.2.1629-1634.1997 8995690 PMC191221

[30] Manska S, Rossetto CC. 2022. Identification of cellular proteins associated with human cytomegalovirus (HCMV) DNA replication suggests novel cellular and viral interactions. Virology (Auckl) 566:26–41. doi:10.1016/j.virol.2021.11.004 PMC8720285 34861458

[31] Huang ES, Huang CH, Huong SM, Selgrade M. 1976. Preferential inhibition of herpes-group viruses by phosphonoacetic acid: effect on virus DNA synthesis and virus-induced DNA polymerase activity. Yale J Biol Med 49:93–98. 960726 PMC2595326

[32] Lurain NS, Chou S. 2010. Antiviral drug resistance of human cytomegalovirus. Clin Microbiol Rev 23:689–712. doi:10.1128/CMR.00009-10 20930070 PMC2952978

[33] Sun Z, Jha HC, Robertson ES. 2015. Bub1 in complex with LANA recruits PCNA to regulate kaposi’s sarcoma-associated herpesvirus latent replication and DNA translesion synthesis. J Virol 89:10206–10218. doi:10.1128/JVI.01524-15 26223641 PMC4580184

[34] Daikoku T, Kudoh A, Sugaya Y, Iwahori S, Shirata N, Isomura H, Tsurumi T. 2006. Postreplicative mismatch repair factors are recruited to Epstein-Barr virus replication compartments. J Biol Chem 281:11422–11430. doi:10.1074/jbc.M510314200 16510450

[35] Sanders I, Boyer M, Fraser NW. 2015. Early nucleosome deposition on, and replication of, HSV DNA requires cell factor PCNA. J Neurovirol 21:358–369. doi:10.1007/s13365-015-0321-7 25672886 PMC4512931

[36] Packard JE, Williams MR, Fromuth DP, Dembowski JA. 2023. Proliferating cell nuclear antigen inhibitors block distinct stages of herpes simplex virus infection. PLoS Pathog 19:e1011539. doi:10.1371/journal.ppat.1011539 37486931 PMC10399828

[37] Hoege C, Pfander B, Moldovan G-L, Pyrowolakis G, Jentsch S. 2002. RAD6-dependent DNA repair is linked to modification of PCNA by ubiquitin and SUMO. Nature 419:135–141. doi:10.1038/nature00991 12226657

[38] Watanabe K, Tateishi S, Kawasuji M, Tsurimoto T, Inoue H, Yamaizumi M. 2004. Rad18 guides poleta to replication stalling sites through physical interaction and PCNA monoubiquitination. EMBO J 23:3886–3896. doi:10.1038/sj.emboj.7600383 15359278 PMC522788

[39] Xu X, Blackwell S, Lin A, Li F, Qin Z, Xiao W. 2015. Error-free DNA-damage tolerance in Saccharomyces cerevisiae. Mutat Res Rev Mutat Res 764:43–50. doi:10.1016/j.mrrev.2015.02.001 26041265

[40] Motegi A, Liaw H-J, Lee K-Y, Roest HP, Maas A, Wu X, Moinova H, Markowitz SD, Ding H, Hoeijmakers JHJ, Myung K. 2008. Polyubiquitination of proliferating cell nuclear antigen by HLTF and SHPRH prevents genomic instability from stalled replication forks. Proc Natl Acad Sci USA 105:12411–12416. doi:10.1073/pnas.0805685105 18719106 PMC2518831

[41] Moldovan G-L, Dejsuphong D, Petalcorin MIR, Hofmann K, Takeda S, Boulton SJ, D’Andrea AD. 2012. Inhibition of homologous recombination by the PCNA-interacting protein PARI. Mol Cell 45:75–86. doi:10.1016/j.molcel.2011.11.010 22153967 PMC3267324

[42] Gali H, Juhasz S, Morocz M, Hajdu I, Fatyol K, Szukacsov V, Burkovics P, Haracska L. 2012. Role of SUMO modification of human PCNA at stalled replication fork. Nucleic Acids Res 40:6049–6059. doi:10.1093/nar/gks256 22457066 PMC3401441

[43] Ertl PF, Powell KL. 1992. Physical and functional interaction of human cytomegalovirus DNA polymerase and its accessory protein (ICP36) expressed in insect cells. J Virol 66:4126–4133. doi:10.1128/JVI.66.7.4126-4133.1992 1318399 PMC241215

[44] Weiland KL, Oien NL, Homa F, Wathen MW. 1994. Functional analysis of human cytomegalovirus polymerase accessory protein. Virus Res 34:191–206. doi:10.1016/0168-1702(94)90124-4 7856311

[45] Strang BL, Boulant S, Chang L, Knipe DM, Kirchhausen T, Coen DM. 2012. Human cytomegalovirus UL44 concentrates at the periphery of replication compartments, the site of viral DNA synthesis. J Virol 86:2089–2095. doi:10.1128/JVI.06720-11 22156516 PMC3302373

[46] Manska S, Octaviano R, Rossetto CC. 2020. 5-Ethynyl-2’-deoxycytidine and 5-ethynyl-2’-deoxyuridine are differentially incorporated in cells infected with HSV-1, HCMV, and KSHV viruses. J Biol Chem 295:5871–5890. doi:10.1074/jbc.RA119.012378 32205447 PMC7196651

[47] Schilling E-M, Scherer M, Rothemund F, Stamminger T. 2021. Functional regulation of the structure-specific endonuclease FEN1 by the human cytomegalovirus protein IE1 suggests a role for the re-initiation of stalled viral replication forks. PLoS Pathog 17:e1009460. doi:10.1371/journal.ppat.1009460 33770148 PMC8026080

[48] Speir E, Shibutani T, Yu Z-X, Ferrans V, Epstein SE. 1996. Role of reactive oxygen intermediates in cytomegalovirus gene expression and in the response of human smooth muscle cells to viral infection. Circ Res 79:1143–1152. doi:10.1161/01.res.79.6.1143 8943952

[49] Tilton C, Clippinger AJ, Maguire T, Alwine JC. 2011. Human cytomegalovirus induces multiple means to combat reactive oxygen species. J Virol 85:12585–12593. doi:10.1128/JVI.05572-11 21937645 PMC3209350

[50] Zlatanou A, Despras E, Braz-Petta T, Boubakour-Azzouz I, Pouvelle C, Stewart GS, Nakajima S, Yasui A, Ishchenko AA, Kannouche PL. 2011. The hMsh2-hMsh6 complex acts in concert with monoubiquitinated PCNA and Pol η in response to oxidative DNA damage in human cells. Mol Cell 43:649–662. doi:10.1016/j.molcel.2011.06.023 21855803

[51] Kashiwaba S, Kanao R, Masuda Y, Kusumoto-Matsuo R, Hanaoka F, Masutani C. 2015. USP7 is a suppressor of PCNA ubiquitination and oxidative-stress-induced mutagenesis in human cells. Cell Rep 13:2072–2080. doi:10.1016/j.celrep.2015.11.014 26673319

[52] Zhang X, Tang N, Xi D, Feng Q, Liu Y, Wang L, Tang Y, Zhong H, He F. 2020. Human cytomegalovirus promoting endothelial cell proliferation by targeting regulator of G-protein signaling 5 hypermethylation and downregulation. Sci Rep 10:2252–2252. doi:10.1038/s41598-020-58680-6 32041970 PMC7010708

[53] Song Y-J, Stinski MF. 2002. Effect of the human cytomegalovirus IE86 protein on expression of E2F-responsive genes: a DNA microarray analysis. Proc Natl Acad Sci USA 99:2836–2841. doi:10.1073/pnas.052010099 11867723 PMC122434

[54] Nitzsche A, Paulus C, Nevels M. 2008. Temporal dynamics of cytomegalovirus chromatin assembly in productively infected human cells. J Virol 82:11167–11180. doi:10.1128/JVI.01218-08 18786996 PMC2573275

[55] Dyson OF, Pagano JS, Whitehurst CB. 2017. The translesion polymerase pol η is required for efficient epstein-barr virus infectivity and is regulated by the viral deubiquitinating enzyme BPLF1. J Virol 91:e00600-17. doi:10.1128/JVI.00600-17 28724765 PMC5599766

[56] Wilkinson GWG, Davison AJ, Tomasec P, Fielding CA, Aicheler R, Murrell I, Seirafian S, Wang ECY, Weekes M, Lehner PJ, Wilkie GS, Stanton RJ. 2015. Human cytomegalovirus: taking the strain. Med Microbiol Immunol 204:273–284. doi:10.1007/s00430-015-0411-4 25894764 PMC4439430

[57] Nightingale K, Lin K-M, Ravenhill BJ, Davies C, Nobre L, Fielding CA, Ruckova E, Fletcher-Etherington A, Soday L, Nichols H, Sugrue D, Wang ECY, Moreno P, Umrania Y, Huttlin EL, Antrobus R, Davison AJ, Wilkinson GWG, Stanton RJ, Tomasec P, Weekes MP. 2018. High-definition analysis of host protein stability during human cytomegalovirus infection reveals antiviral factors and viral evasion mechanisms. Cell Host Microbe 24:447–460. doi:10.1016/j.chom.2018.07.011 30122656 PMC6146656

[58] Unk I, Hajdú I, Fátyol K, Hurwitz J, Yoon J-H, Prakash L, Prakash S, Haracska L. 2008. Human HLTF functions as a ubiquitin ligase for proliferating cell nuclear antigen polyubiquitination. Proc Natl Acad Sci USA 105:3768–3773. doi:10.1073/pnas.0800563105 18316726 PMC2268824

[59] Zarrella K, Longmire P, Zeltzer S, Collins-McMillen D, Hancock M, Buehler J, Reitsma JM, Terhune SS, Nelson JA, Goodrum F. 2023. Human cytomegalovirus UL138 interaction with USP1 activates STAT1 in infection. PLoS Pathog 19:e1011185. doi:10.1371/journal.ppat.1011185 37289831 PMC10284425

[60] Whitehurst CB, Vaziri C, Shackelford J, Pagano JS. 2012. Epstein-Barr virus BPLF1 deubiquitinates PCNA and attenuates polymerase η recruitment to DNA damage sites. J Virol 86:8097–8106. doi:10.1128/JVI.00588-12 22623772 PMC3421674

[61] Dong X, Guan J, Zheng C, Zheng X. 2017. The herpes simplex virus 1 UL36USP deubiquitinase suppresses DNA repair in host cells via deubiquitination of proliferating cell nuclear antigen. J Biol Chem 292:8472–8483. doi:10.1074/jbc.M117.778076 28348081 PMC5437251

[62] Wang J, Loveland AN, Kattenhorn LM, Ploegh HL, Gibson W. 2006. High-molecular-weight protein (pUL48) of human cytomegalovirus is a competent deubiquitinating protease: mutant viruses altered in its active-site cysteine or histidine are viable. J Virol 80:6003–6012. doi:10.1128/JVI.00401-06 16731939 PMC1472576

[63] Weaver TM, Click TH, Khoang TH, Todd Washington M, Agarwal PK, Freudenthal BD. 2022. Mechanism of nucleotide discrimination by the translesion synthesis polymerase Rev1. Nat Commun 13:2876. doi:10.1038/s41467-022-30577-0 35610266 PMC9130138

[64] Ohashi E, Murakumo Y, Kanjo N, Akagi J, Masutani C, Hanaoka F, Ohmori H. 2004. Interaction of hREV1 with three human Y‐family DNA polymerases. Genes Cells 9:523–531. doi:10.1111/j.1356-9597.2004.00747.x 15189446

[65] Guo C, Sonoda E, Tang T-S, Parker JL, Bielen AB, Takeda S, Ulrich HD, Friedberg EC. 2006. REV1 protein interacts with PCNA: significance of the REV1 BRCT domain in vitro and in vivo. Mol Cell 23:265–271. doi:10.1016/j.molcel.2006.05.038 16857592

[66] Thakar T, Leung W, Nicolae CM, Clements KE, Shen B, Bielinsky AK, Moldovan GL. 2020. Ubiquitinated-PCNA protects replication forks from DNA2-mediated degradation by regulating Okazaki fragment maturation and chromatin assembly. Nat Commun 11:2147. doi:10.1038/s41467-020-16096-w 32358495 PMC7195461

[67] Umashankar M, Rak M, Bughio F, Zagallo P, Caviness K, Goodrum FD. 2014. Antagonistic determinants controlling replicative and latent states of human cytomegalovirus infection. J Virol 88:5987–6002. doi:10.1128/JVI.03506-13 24623432 PMC4093889

