# Supplementary material for: Complex roles for proliferating cell nuclear antigen in restricting human cytomegalovirus replication
Source: mBio. 2025 Mar 25;16(5):e00450-25. doi: 10.1128/mbio.00450-25 (PMC12077088; doi:10.1128/mbio.00450-25)
Supplement: Supplemental material — Fig. S1 and extended materials and methods. [file mbio.00450-25-s0001.docx]

**SUPPLEMENTAL MATERIAL**


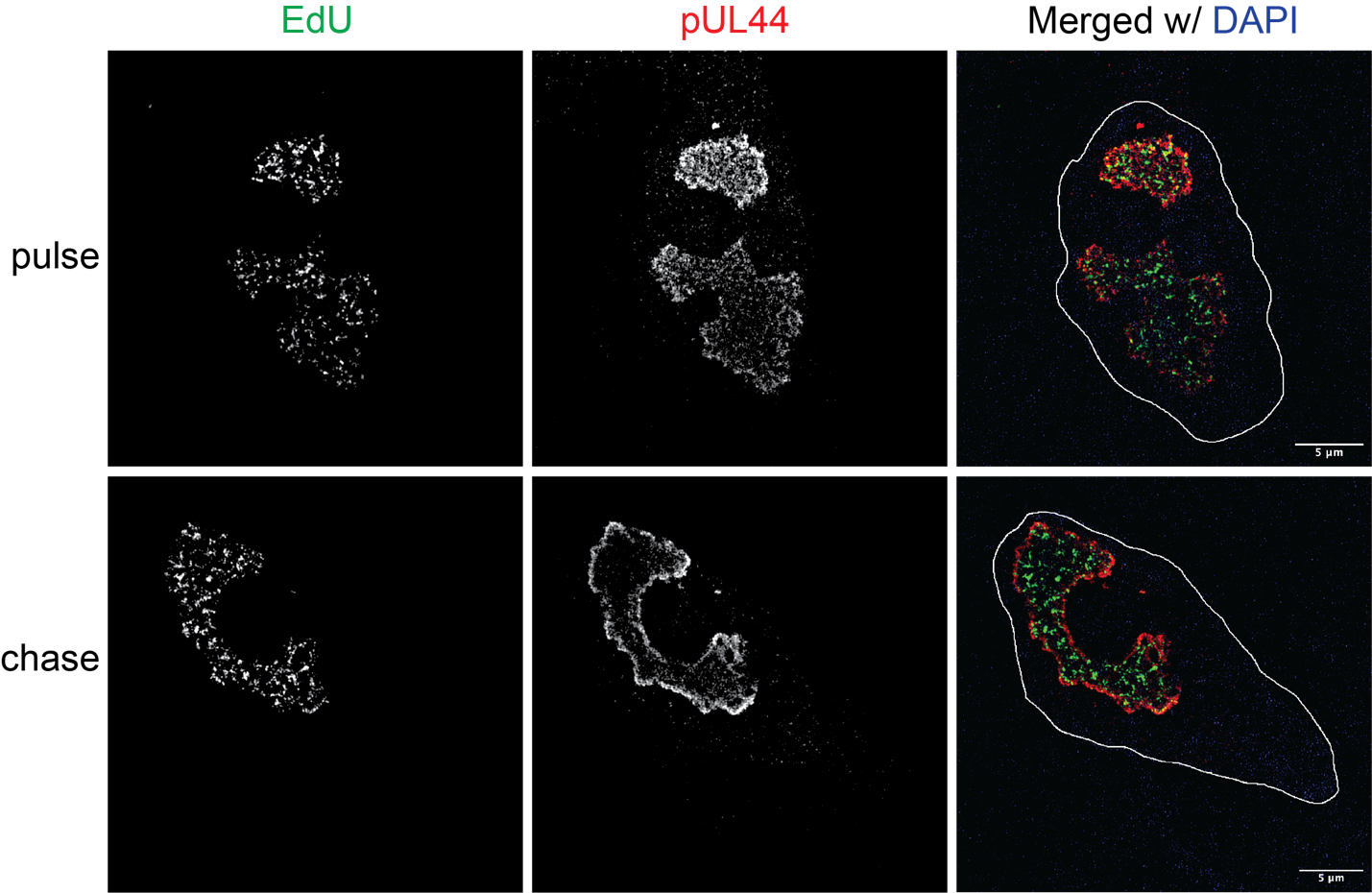


**Figure S1. EdU pulse-chase labeling of viral replication compartments in HCMV AD169 infection.**

Fibroblasts were serum-starved and then infected with AD169-WT at an MOI of 1. At 48 hpi, half of the cells were pulsed with 10 µM EdU for 10 minutes, CSK-extracted, and fixed. Following EdU pulse, the other half of the cells were incubated with 200 µM thymidine for one hour and subsequently CSK-extracted and fixed. All coverslips were washed and then a click reaction was performed to conjugate EdU to Alexa Fluor 647 (shown in green) for detection. Indirect immunofluorescence was then carried out using monoclonal antibodies to pUL44 for detection of viral RCs with a secondary antibody conjugated to Alexa Fluor® 488 (shown in red). DAPI-stained nuclei (individual images not shown) were outlined using Fiji/ImageJ software. Images were obtained using a Zeiss Elyra S.1 super-resolution microscope. Scale bar, 5 µm.

SUPPLEMENTAL MATERIALS AND METHODS

**EdU pulse labeling.** Fibroblasts were seeded onto 12-mm 1.5H high precision coverslips (Marienfeld Superior) in 24-well plates in DMEM containing 10% FBS. The next day, cells were washed three times in PBS and growth medium was replaced with serum-free (0% FBS) culture medium. On the following day, cells were infected with HCMV TB40/E at an MOI of 1. At 48 hours post-infection, half of the media was replaced with serum-free DMEM containing 20 µM EdU for a final concentration of 10 µM EdU. Cells were then incubated at 37ºC with 5% CO_2_. After 10 minutes, the coverslips were washed in PBS and then incubated in cold cytoskeletal (CSK) extraction buffer for two minutes (1). Following CSK extraction, cells were fixed with 100% methanol for 10 minutes at -20ºC. After fixation, cells were washed twice with PBS containing 3% BSA. For detection of EdU, the click reaction was performed according to manufacturer instructions (Invitrogen #C10340). Indirect immunofluorescence was subsequently performed as described below.

**Immunofluorescence and super-resolution microscopy.** Fibroblasts were seeded onto 12-mm 1.5H high precision coverslips (Marienfeld Superior) in 24-well plates. After EdU pulse labeling (described above), coverslips were processed for indirect immunofluorescence as previously described (2). Briefly, proteins of interest were detected using specific primary antibodies for one hour at room temperature or overnight at 4ºC as described in Table 1. Coverslips were washed 3 times in PBS + 0.05% Tween20 and then incubated in secondary antibodies (AlexaFluor 546, AlexaFluor 647, or AlexaFluor 488 goat anti-mouse or goat anti-rabbit [Invitrogen]) for 30 minutes at room temperature. Coverslips were incubated in DAPI for 5 minutes and then washed three times in PBS + 0.05% Tween20. Finally, coverslips were mounted onto microscope slides (Fisher Scientific) using Prolong Gold Antifade Mounting Reagent (Invitrogen). Images were obtained using a Zeiss Elyra S.1 super-resolution microscope using a Zeiss 63x Plan-Apochromat 1.40NA objective with structured illumination (SR-SIM) processing. Representative single plane images were adjusted for brightness and contrast using Fiji/ImageJ software.

**Colocalization analysis of host and viral proteins to EdU staining in host cell nuclei.** Nikon NIS Elements AR 5.42.03 software with the General Analysis 3 (GA3) module was used for image processing and analysis. The following image processing tools were used to achieve accurate segmentation on the EdU, host protein, and viral protein foci channels: Rolling ball background subtraction (radius = 15px) and Median filter (kernel size = 1px). The Bright Spots Detection tool (diameter = 2px) was used to threshold the EdU, Host, and Viral Protein foci. For DAPI, a Gaussian filter (sigma = 8), rolling ball background subtraction (radius = 220px), and gamma correction (gamma = 0.8) were applied. A Signal Intensity threshold was used to threshold host nuclei and the resulting nuclei binary objects were then modified using Grow Regions and Smooth tools to further refine nuclear segmentation. The AND binary operation was performed between DAPI and corresponding EdU, host protein, and viral protein foci to restrict analysis to only the foci present inside each cell nucleus. To quantify the frequency of colocalization between EdU/Host Foci and EdU/Viral foci, respectively, the AND binary operation was performed between these two paired groups and the Object Count function was used to quantify each group.

**Genomic sequencing and computational analysis.** Fibroblasts were seeded onto 6-cm dishes and transduced with lentiviral constructs as described above. Five technical replicates were seeded for each condition. At four days post transduction, cells were infected with HCMV-TB40/E at an MOI of 1. Virus inoculum was removed at 2 hpi and cells were provided fresh media. At 96 hpi, when maximal cytopathic effect (CPE) was observed, cells were washed with PBS and collected in DNA lysis buffer containing 200 µg/mL proteinase K by manual scraping. After a two hour 55ºC incubation for proteinase K digestion, cellular and viral DNA were isolated using phenol-chloroform extraction. DNA was similarly extracted from the virus stock used for infection. All purified DNA was submitted to SeqCenter (Pittsburgh, PA).

Each sample (including virus stocks) was sequenced on a NextSeq 2000 Illumina short read sequencer to yield paired-end short reads. The sequencing reads were aligned to the human reference genome GRCh38 (3) using Bowtie2 v2.5.1 (4) with the following parameters: --very-sensitive for sensitive alignment, --seed 1 for seeding alignment. After alignment, reads aligned to the human genome were filtered out using Samtools v1.17 (5). The following parameters were used: -f UNMAP,MUNMAP to extract reads that were not aligned to the human genome, and -bh to output the filtered alignments in BAM format. HCMV junctions and SNVs were then detected using a two-pass analysis to ensure comparable sequencing coverage between the samples. Non-human reads were first aligned to the reference HCMV genome with breseq v0.38.1 (6) using polymorphism-prediction mode. Using the mean sequencing coverage of reads aligned to the reference from the first breseq run output, the sequencing reads from each sample were subsampled with seqtk v1.4q (https://github.com/lh3/seqtk) using parameter -s 100 and the appropriate respective proportions to yield the same mean coverage equal to that of the sample with the lowest coverage (shRNA-Luc). The subsampled FASTQ files were then used to detect the junctions and SNVs by running breseq in the --polymorphism-prediction mode again. Subsequent data analyses were conducted in R version 4.3.2 (7). The novel junctions and SNVs were obtained by removing any of those found in the virus stock samples from the total junctions and SNVs detected in each experimental sample. A Poisson two-sided test was used to compare junction frequency across experimental conditions, with a junction frequency cutoff of 0.025 employed to refine the selection of relevant sequences. Additionally, circle plots were constructed using ‘circlize’ (version 0.4.16) (8) to visualize the locations and relationships of these sequences, providing a comprehensive view of their distribution and interaction within the genome. This multifaceted approach allowed for a nuanced exploration of genomic junctions, highlighting significant variations and patterns across experimental conditions.

**REFERENCES**

1. Dimitrova DS, Gilbert DM. 2000. Stability and Nuclear Distribution of Mammalian Replication Protein A Heterotrimeric Complex. Experimental cell research 254:321-327.

2. Zeltzer S, Longmire P, Svoboda M, Bosco G, Goodrum F. 2022. Host translesion polymerases are required for viral genome integrity. Proceedings of the National Academy of Sciences - PNAS 119:e2203203119-e2203203119.

3. Schneider VA, Graves-Lindsay T, Howe K, Bouk N, Chen H-C, Kitts PA, Murphy TD, Pruitt KD, Thibaud-Nissen F, Albracht D, Fulton RS, Kremitzki M, Magrini V, Markovic C, McGrath S, Steinberg KM, Auger K, Chow W, Collins J, Harden G, Hubbard T, Pelan S, Simpson JT, Threadgold G, Torrance J, Wood JM, Clarke L, Koren S, Boitano M, Peluso P, Li H, Chin C-S, Phillippy AM, Durbin R, Wilson RK, Flicek P, Eichler EE, Church DM. 2017. Evaluation of GRCh38 and de novo haploid genome assemblies demonstrates the enduring quality of the reference assembly. Genome research 27:849-864.

4. Langmead B, Salzberg SL. 2012. Fast gapped-read alignment with Bowtie 2. Nature methods 9:357-359.

5. Li H, Handsaker B, Wysoker A, Fennell T, Ruan J, Homer N, Marth G, Abecasis G, Durbin R. 2009. The Sequence Alignment/Map format and SAMtools. Bioinformatics 25:2078-2079.

6. Deatherage DE, Barrick JE. 2014. Identification of Mutations in Laboratory-Evolved Microbes from Next-Generation Sequencing Data Using breseq.165-188.

7. Team RC. 2023. R: A Language and Environment for Statistical Computing. R Foundation for Statistical Computing, Vienna, Austria.

8. Gu Z, Gu L, Eils R, Schlesner M, Brors B. 2014. circlize Implements and enhances circular visualization in R. Bioinformatics (Oxford, England) 30:2811-2812.
